# Linkages between soil nutrient turnover and above‐ground crop nutrient metabolism: The role of soil microbes

**DOI:** 10.1002/imo2.55

**Published:** 2025-02-03

**Authors:** Wensheng Fang, Wenfeng Tian, Dongdong Yan, Yuan Li, Aocheng Cao, Qiuxia Wang

**Affiliations:** ^1^ State Key Laboratory for Biology of Plant Diseases and Insect Pests, Institute of Plant Protection Chinese Academy of Agricultural Sciences Beijing China; ^2^ Horticulture College Hunan Agricultural University Changsha China

**Keywords:** fumigant dozamet, nitrogen metabolism, nutrient turnover, soil fumigation, soil microorganism

## Abstract

With global agricultural practices increasingly focused on sustainability and efficiency, deciphering the complex relationship between soil nutrient turnover and crop nutrient uptake is critical for advancing crop productivity and environmental resilience. This study focuses on the nitrogen transformation process following dazomet soil fumigation and examines how soil microorganisms influence both soil nitrogen cycling and above‐ground crop nitrogen metabolism. Fumigation led to significant changes, with ammonium nitrogen increasing by 18.89%−94.82% and nitrate nitrogen decreasing by 30.20%−90.21% in tobacco roots, stems, and leaves. Fumigation also caused a notable shift in the soil microbial community, inhibiting ammonium‐oxidizing microorganisms while stimulating denitrifying microbes. These changes not only disrupted the nitrogen balance‐reducing soil nitrification by 72.91%−86.51% and increasing denitrification by 197%−324% but also had a cascading effect on above‐ground crop nitrogen metabolism. By altering the composition of microbial communities, the process directly influenced soil nutrient turnover, subsequently impacting nutrient availability and distribution in crop. The disruption in nitrogen balance further led to changes in the expression of key nitrogen metabolism enzymes and transporter genes. Specifically, genes related to nitrate and ammonium transporters, as well as amino acid and nitrogen metabolism enzymes, were upregulated. Structural equation modeling confirmed that the shifts in the microbial community were central to driving changes in both soil nutrient turnover and nitrogen distribution in the crop. These findings underscore the critical role of soil microbial communities as a link between soil nutrient cycling and above‐ground nutrient metabolism, highlighting their importance in regulating plant nutrient absorption and utilization. This suggests that optimizing microbial management strategies could lead to significant improvements in crop nutrient efficiency.

## Highlights

- Fumigation significantly alters the spatial distribution of nitrogen in plants.
- Fumigation drastically shifts soil microbial communities by promoting denitrifying microbes.
- The changes in microbial communities lead to a disruption of soil nitrogen balance.
- The activities of metabolic enzymes and nitrogen transporter gene expressions are stimulated.
- Soil microorganisms influence above‐ground plant nutrient metabolism by modifying nutrient turnover processes.

## Graphical abstract

Fumigation significantly impacts soil nitrogen dynamics and above‐ground nutrient metabolism in plants by altering microbial community structure and nitrogen forms. This process increases ammonium nitrogen while reducing nitrate nitrogen levels in plant tissues, a shift driven by the suppression of ammonium‐oxidizing microbes and the promotion of denitrifying microbes in soil. Such microbial community changes disrupt the soil nitrogen balance, influencing the availability and distribution of nitrogen to plants. The altered nitrogen profile in subsurface soil subsequently modulates the activities of key metabolic enzymes and the expression of nitrogen transporter genes in above‐ground tissues, highlighting the role of soil microorganisms in regulating nutrient turnover and above‐ground nutrient metabolism.

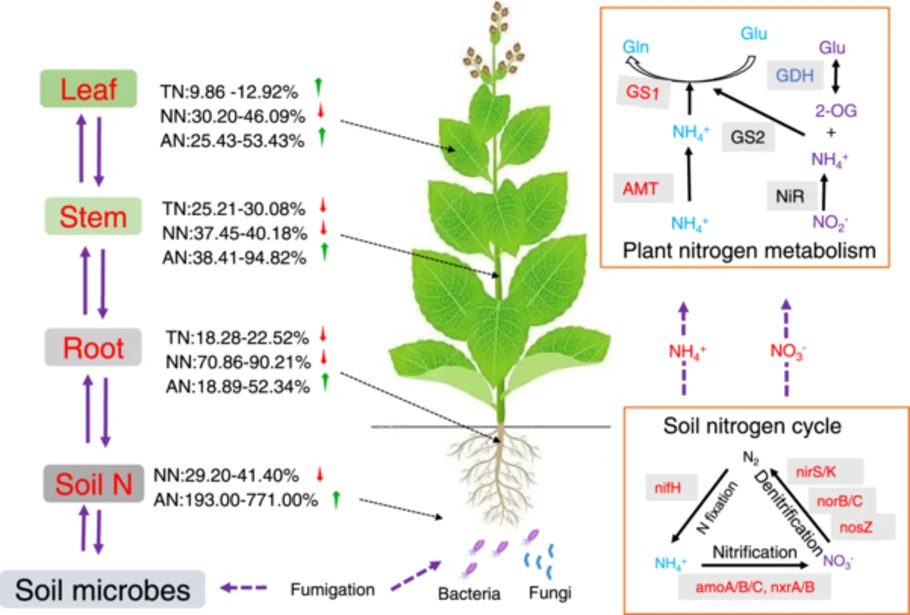

## 1 INTRODUCTION

The interaction between soil microbes and crops is a complex and dynamic process within ecosystems, involving material circulation, energy flow, and information transfer [1, 2, 3, 4]. This intricate interplay is essential for maintaining ecological balance and productivity. Despite significant advancements in scientific research, many unanswered questions persist, particularly regarding how soil microorganisms strongly regulate soil nutrient turnover and influence the distribution of nutrient elements within crop [5, 6, 7]. Understanding these interactions is crucial for uncovering the fundamental mechanisms of ecosystem functions and enhancing agricultural productivity, ultimately contributing to sustainable environmental management and food security.

Soil fumigation is a well‐established soil treatment method that significantly improves crop growing conditions by targeting and eradicating pathogenic microorganisms, including fungi, bacteria, and nematodes [8, 9, 10]. This technique is particularly effective in managing soil‐borne diseases, thus safeguarding crop health and enhancing yield potential. Additionally, soil fumigation provides a notable “fertilizer effect” by enhancing overall soil health, which facilitates improved nutrient and water uptake by crops, promoting more vigorous growth and higher yields [11, 12, 13]. However, empirical studies indicate that soil fumigation can disrupt the nitrogen metabolism, leading to significant changes in the presence form and quantity of inorganic nitrogen in soil [14, 15, 16, 17, 18]. The impact of these disruptions on crop nitrogen uptake and the spatial distribution of nitrogen within plant tissues remains poorly understood. Therefore, further research is crucial to elucidate the comprehensive effects of soil fumigation on nitrogen metabolism and to optimize its application to achieve better agricultural outcomes.

Soil microorganisms play a pivotal role in the interactions between nutrient turnover and crop metabolism. They drive the decomposition of organic matter and mineralization processes, converting complex organic compounds into essential inorganic nutrients such as nitrogen, phosphorus, and potassium that crops can readily absorb [19, 20]. This microbial activity is crucial for crop health, influencing nutrient availability and metabolism in the above‐ground parts of crops. Key microorganisms, including nitrogen‐fixing bacteria and mycorrhizal fungi, significantly enhance nutrient uptake, thus directly impacting crop growth and productivity [21, 22]. The interaction between soil nutrients and crops is mediated through root exudates, which are compounds released by crop roots into the soil. These exudates provide a carbon and energy source for soil microorganisms, stimulating their growth and activity [23, 24]. This mutualistic relationship promotes efficient nutrient cycling and availability, thereby supporting crop nutrient uptake and metabolism. The continuous exchange of nutrients between crops and microorganisms ensures that essential elements are effectively mobilized and utilized, optimizing crop health and productivity. Furthermore, by altering nutrient availability and cycling processes, microorganisms regulate the supply of essential nutrients to crops [25]. This interaction highlights the importance of managing soil microbial communities to enhance nutrient turnover and improve crop nutrient absorption. Effective management practices can lead to more efficient nutrient utilization, supporting sustainable agricultural practices and improving crop yields. This integrated view of soil microbiota, nutrient dynamics, and crop metabolism underscores the critical role of soil microorganisms in maintaining soil fertility and crop health [26, 27].

Soil fumigant dazomet (DZ), widely used in China, decomposes rapidly into methyl isothiocyanate, its active component, which facilitates its role in fumigation [28]. This study investigates the effects of DZ fumigation on nitrogen metabolism, specifically examining how it influences nitrogen uptake and spatial distribution in crops. The objectives are twofold: (1) to elucidate the spatial distribution of nitrogen in crops and the regulatory mechanisms involved following fumigation with DZ and (2) to explore the potential mechanisms by which soil microorganisms affect the distribution of above−ground nutrients in crops. The results will provide critical insights into the interactions among soil nutrients, microorganisms, and crops, thereby offering a theoretical foundation for enhancing agricultural practices and advancing ecological sustainability.

## 2 RESULTS

### Temporal and spatial variation of nitrogen dynamics under DZ fumigation

**FIGURE 1:**
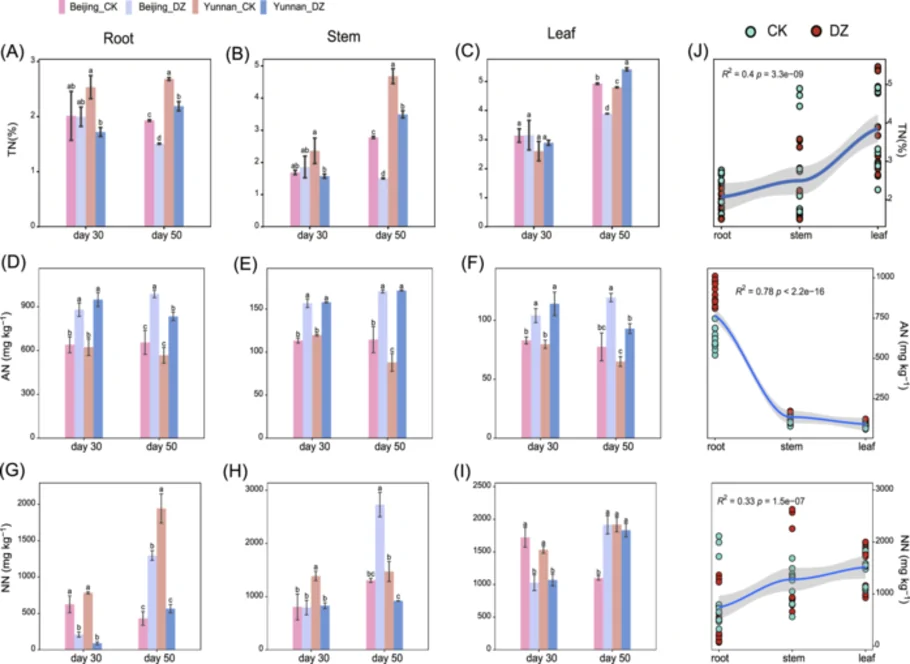
Spatial and temporal distribution of total nitrogen (TN), ammonium nitrogen (AN), and nitrate nitrogen (NN). Changes in TN in roots (A), stems (B) or leaves (C). Changes in AN in roots (D), stems (E), or leaves (F). Changes in NN in roots (G), stems (H), or leaves (I). The significance of the groups is indicated by lowercase letters, with different letters representing significant differences. (J) Spearman correlation between the three nitrogen forms and spatial distribution.

In Beijing soil, DZ fumigation did not alter total nitrogen (TN) content in roots, stems, and leaves by Day 30 (Figure 1A−C). By Day 50, significant reductions were evident: roots (21.89% decrease, *p* < 0.05), stems (45.81% decrease, *p* < 0.05), and leaves (20.95% decrease, *p* < 0.05) compared to the non‐fumigation group (Figure 1A−C). Conversely, in Yunnan soil, nitrogen content in roots and stems showed consistent decreases in the fumigation groups on Days 30 (32.11%−33.29% reduction, *p* < 0.05) and 50 (18.44%−25.35% reduction, *p* < 0.05) (Figure 1A−C). Leaves initially showed no difference at Day 30 but exhibited significantly higher nitrogen content by Day 50 in the fumigation group compared to unfumigated group (12.92% increase, *p* < 0.05). These findings indicate that DZ fumigation leads to a progressive depletion of nitrogen in Beijing soil over time. In contrast, in Yunnan soil, the impact of fumigation is more immediate, peaking early with a subsequent decline in nitrogen levels in both Beijing and Yunnan soils, ammonium nitrogen (AN) content in roots, stems, and leaves was markedly elevated in response to DZ fumigation compared to controls (increases of 37.49%−52.34%, 31.91%−94.82%, and 25.44%−53.96%, respectively; all *p* < 0.05, Figure 1D−F). Notably, in Yunnan soil, the increase in AN in stems was significantly higher by Day 50 compared to Day 30 (94.82% vs. 31.91%, *p* < 0.05) (Figure 1E). Similarly, in Beijing soil, the increases in roots (50.68% vs. 37.49%, *p* < 0.05), stems (48.89% vs. 38.14%, *p* < 0.05), and leaves (53.96% vs. 25.44%, *p* < 0.05) by Day 50 were significantly higher compared to Day 30. These results underscore the significant temporal and spatial variations influenced by soil type following DZ fumigation.

In Yunnan soil, DZ fumigation significantly stimulated nitrate nitrogen (NN) metabolism in roots, stems, and leaves, resulting in reductions of 70.85%−88.64%, 47.45%−40.18%, and 4.5%−30.20%, respectively (all *p* < 0.05 except leaves on Day 50, Figure 1G−I). This effect was most pronounced on Day 30, particularly in roots, followed by stems and leaves. Conversely, in Beijing soil, the promotion of NN metabolism was primarily observed on Day 30 (reduction of 66.39% in roots, *p* < 0.05; negligible change in stems and reduction of 40.28% in leaves, *p* < 0.05). By Day 50, the fumigation effect transitioned to inhibition, resulting in significant increases in roots (200.43%), stems (108.74%), and leaves (74.09%) (all *p* < 0.05). Spatial analysis revealed a sharp decline in AN levels in roots, stems, and leaves following DZ fumigation or not, while TN and NN exhibited gradual increases, predominantly accumulating in the leaves (Figure 1J).

### Nitrogen‐enzyme dynamics in different compartments following DZ fumigation

**FIGURE 2:**
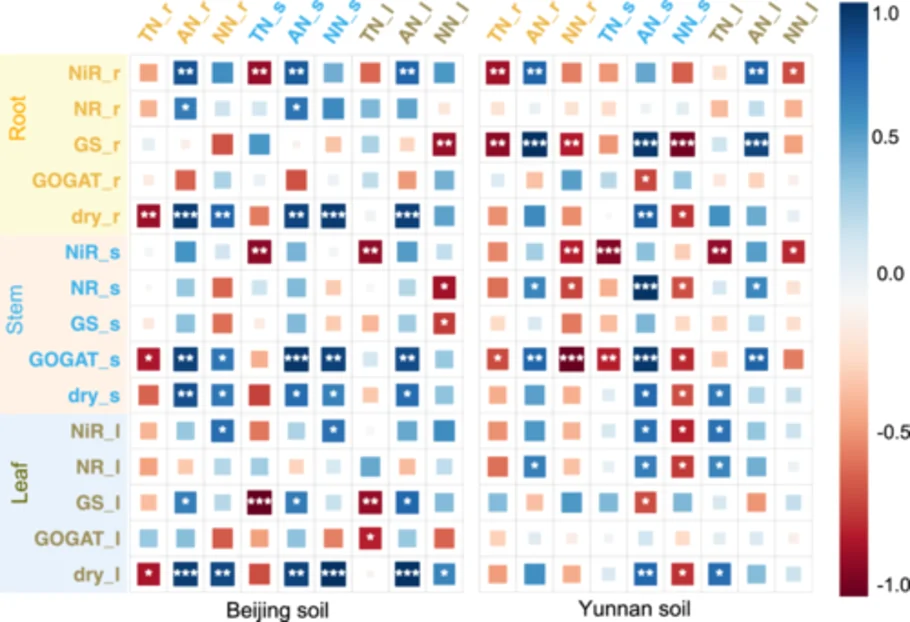
Heatmaps illustrating the correlation between the total nitrogen (TN), ammonium nitrogen (AN), and nitrate nitrogen (NN) and the activity matrix of tobacco roots, stems, and leaves in Beijing soil and Yunnan. Blue indicates a positive correlation, while red indicates a negative correlation. Asterisks within the boxes denote significance levels: *p* < 0.05, *p* < 0.01, *p < 0.001.

In both Beijing and Yunnan soils, reductions in total nitrogen (TN) correlated consistently with elevated activities of nitrite reductase (NiR), glutamine synthetase (GS), and glutamate synthase (GOGAT) across roots, stems, and leaves (Figure 2), suggesting that soil fumigation‐induced these enzymatic activities stimulate TN reductions. Additionally, elevated levels of ammonium nitrogen (AN) in both soil types were associated with enhanced activities of NiR, nitrate reductase (NR), GS, and GOGAT (Figure 2), promoting the upregulation of these enzymes and facilitating nutrient assimilation to enhance plant growth. However, nitrate nitrogen (NN) responses varied significantly between the two soils: in Beijing soil, NN positively correlated with dry weight and GOGAT activity, whereas in Yunnan soil, NN showed negative correlations with certain enzymes (GS, NR, GOGAT) (Figure 2), underscoring soil−specific regulatory mechanisms affecting nitrogen utilization and enzyme activity. Overall, soil fumigation significantly influences the dynamics of nitrogen‐enzyme activities in tobacco. The increased enzymatic activity is consistently associated with reductions in TN and increases in AN. However, the response of NN varies depending on the soil type, highlighting the complex interplay between soil characteristics, nitrogen forms, and enzyme activities.

### Soil nitrogen dynamics and microbial response following DZ fumigation

**FIGURE 3:**
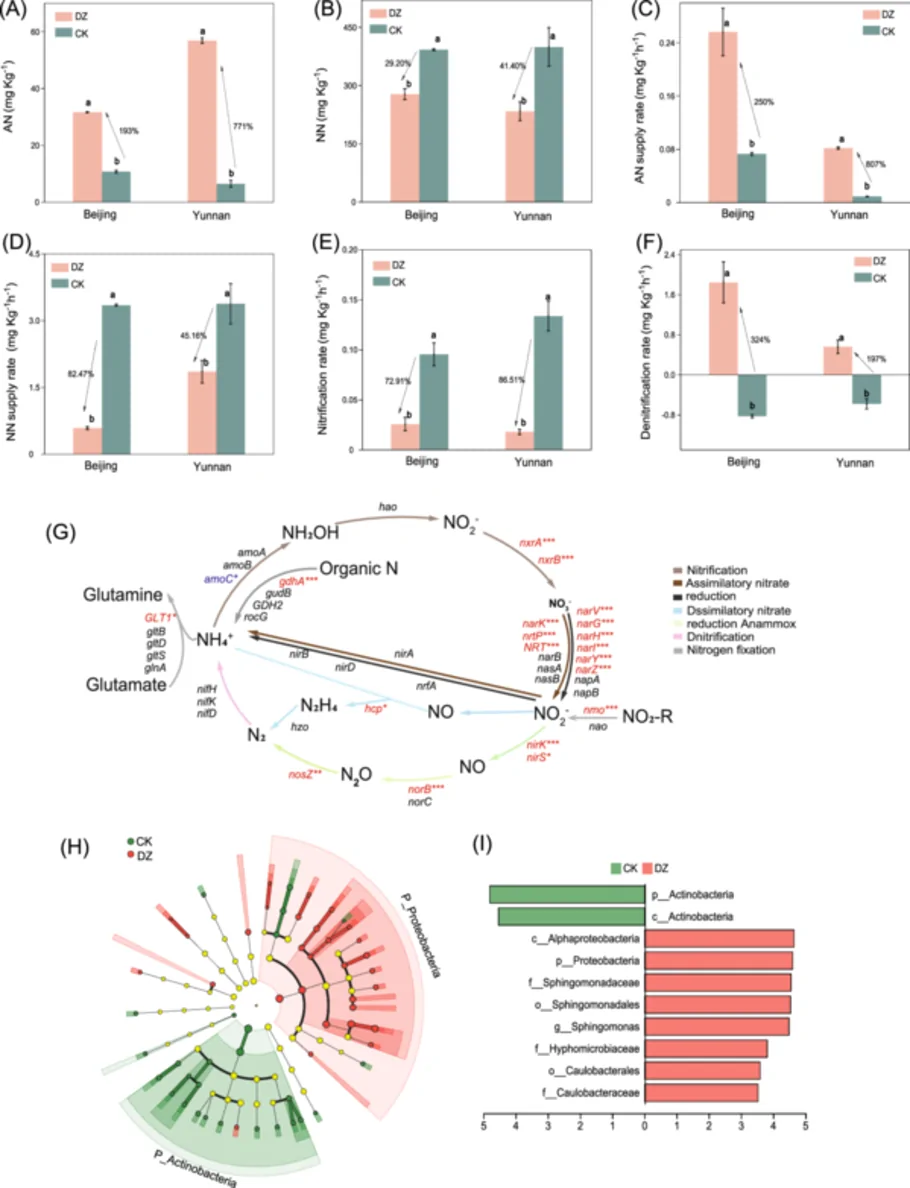
Characteristics of soil nitrogen transformation and microbial response on the 50th day. (A) Ammonium nitrogen (AN), (B) Nitrate nitrogen (NN), (C) Rhizosphere ammonium nitrogen supply rate, (D) Rhizosphere nitrate nitrogen supply rate, (E) Rhizosphere soil nitrification rate, (F) Rhizosphere soil denitrification rate. (G) Overview of functional gene changes related to the nitrogen cycle pathway. Colored lines represent different pathways, with genes responsible for each pathway marked along the lines. Gene names are labeled red for significant up‐regulation compared to the control, blue for significant down‐regulation, and black for no significant change. Asterisks indicate the significance level. (H) LEfSe multi‐level species hierarchy diagram. Different colored nodes indicate microbial groups significantly enriched in the respective treatment group, contributing to group differences. Light yellow nodes represent microbial groups with no significant difference or effect between groups. (I) Linear discriminant analysis (LDA) discriminant histogram. Higher LDA scores indicate a greater influence of species abundance on the differential effect. LEfSe, Linear discriminant analysis Effect Size.

DZ fumigation had a significant impact on nitrogen dynamics in both Beijing and Yunnan soils, increasing AN and decreasing NN, with effects persisting for 50 days (Figure 3 & Figure S2). On the 50th day, the AN levels in fumigated soils increased by 193%−771% (*p* < 0.05) (Figure 3A), while NN levels decreased by 29.20%−41.40% (*p* < 0.05) (Figure 3B), more prominently in Yunnan soil. These changes led to substantial shifts in nitrogen supply and conversion within the rhizosphere. Specifically, AN supply rates surged by 250% in Beijing and 807% in Yunnan (both *p* < 0.05), while NN supply rates significantly declined by 82.47% and 45.16%, respectively (*p* < 0.05) (Figure 3C,D). Consequently, the potential of nitrification decreased by 72.91%−86.51% (*p* < 0.05) (Figure 3E), and the potential of denitrification increased by 197%−324% (*p* < 0.05) (Figure 3F). These findings are consistent with previous research, which has shown that fumigation strongly inhibits nitrification while enhancing denitrification [16, 17, 29].

Immediately following fumigation (Day 0), there was a significant increase in both microbial community diversity and KEGG‐based functional diversity (Figure S1A). However, by the late fumigation period (Day 50), no significant differences were observed between the fumigation and control groups in these diversity measures (Figure S1B). Nonetheless, PCA results indicated distinct differentiation in species classification and functional gene composition (Figure S1C & S1D), suggesting that fumigation selectively enriched specific species and functional pathways.

In DZ‐fumigated Yunnan soil, major nitrogen transformation processes, including nitrification, denitrification, nitrate reduction, anammox, N_2_ fixation, and organic nitrogen metabolism, were analyzed (Figure 3G & Figure S2C). Initially, only denitrification was significantly stimulated, increasing the abundance of *nirS* and *norB* genes (Figure S2C & Table S3). By Day 50, most genes involved in denitrification, such as *nar* family, *nirK/S*, *norB*, and *nosZ*, showed significant increases, while ammonia oxidation genes like *amoC* decreased (Figure 3G). These genes abundance corresponded with observed soil nitrification and denitrification rates (Figure 3E,F). These findings align with previous research, which demonstrates that fumigation‐induced promotion of denitrifying microorganisms directly contributes to increased nitrous oxide emissions [16].

Proteobacteria were notably enriched in fumigated soils for 50 days (Figures S2D & Figure 3H), with significant increases in denitrifying taxa such as Sphingomonadales, Caulobacterales, and Alphaproteobacteria (Figure 3I). For instance, Hyphomicrobiaceae increased by 127% (*p* < 0.05), Caulobacteraceae by 150% (*p* < 0.05), and Sphingomonadaceae by 113% (*p* < 0.05) (Figure S2G). Additionally, *Sphingomonas* increased by 43.81% (*p* < 0.05) on Day 0 and 147% (*p* < 0.05) on Day 50 post‐fumigation, indicating a sustained stimulatory effect on this genus (Figure S2F & S2G).

### Gene expression of nitrogen metabolism in crop following DZ fumigation

**FIGURE 4:**
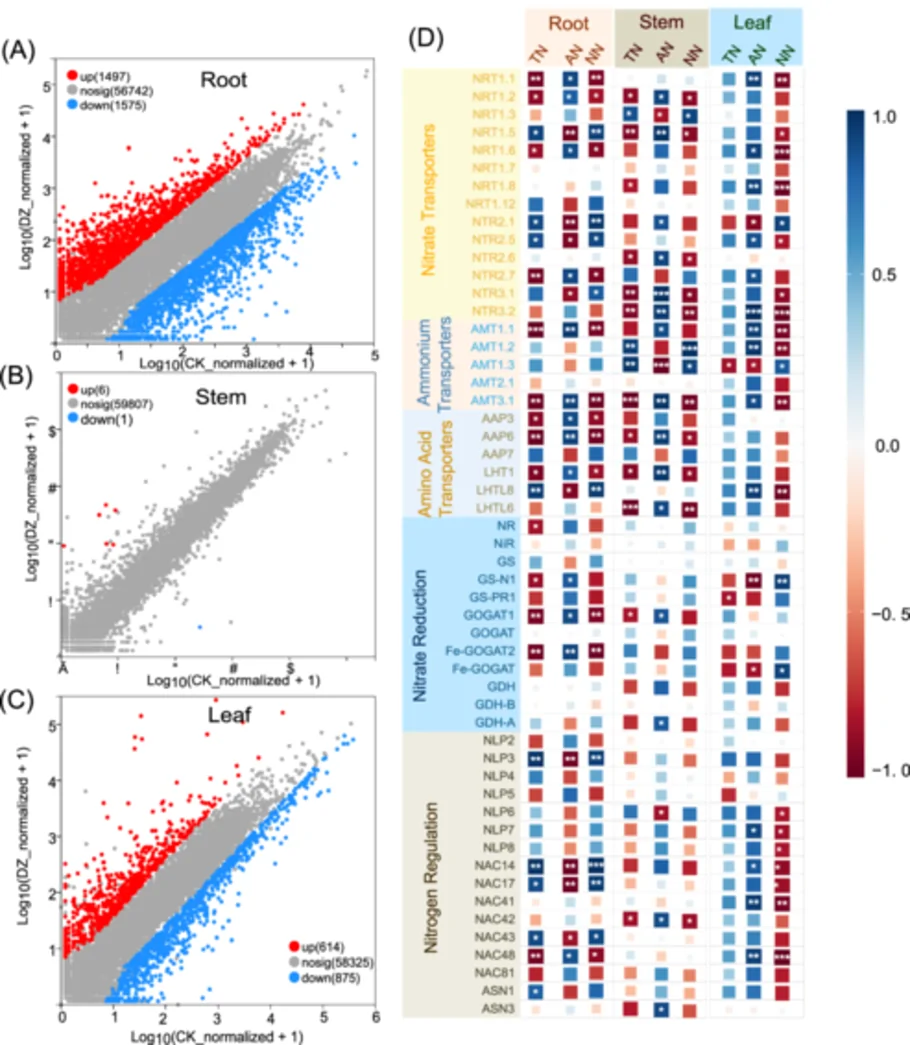
Gene expression characteristics. The scatter plot shows gene expression differences in root (A), stem (B), and leaf (C), with log‐transformed expression levels in control and treatment groups on the horizontal and vertical axes, respectively. Red dots indicate significantly up‐regulated genes, green dots indicate significantly down‐regulated genes, and gray dots indicate non‐significantly different genes. Points closer to the origin represent lower expression levels, while points farther from the diagonal indicate greater differences in gene expression between the two samples. (D) Heatmaps illustrate the correlation between the total nitrogen (TN), ammonium nitrogen (AN), and nitrate nitrogen (NN) of roots, stems, or leaves and nitrogen metabolism gene expression matrix in Yunnan soil. Blue indicates a positive correlation, while red indicates a negative correlation. Asterisks within the boxes denote significance levels: *p* < 0.05, *p* < 0.01, *p < 0.001.

Compared to stems, roots, and leaves are enriched with a larger number of functionally specific metabolic pathways. For example, roots are significantly enriched in pathways such as plant‐type cell wall biogenesis, cellulose metabolic process, xylan biosynthetic process, lignin catabolic process, phenylpropanoid catabolic process, plant‐type secondary cell wall biogenesis, and plant‐type primary cell wall biogenesis (Figure S3A). Leaves, on the other hand, are mainly enriched in pathways related to the photosystem II oxygen‐evolving complex, regulation of photosynthesis, porphyrin‐containing compound biosynthetic process, photosynthesis, and light harvesting (Figure S3C). As a result, fumigation caused 1497 genes to be significantly upregulated in the roots and 614 in the leaves, while only six genes were significantly upregulated in the stems (Figure 4A−C). These results indicate that the roots are a functional hotspot in crops, with fumigation having the greatest impact on gene expression in the roots, followed by the leaves and stems. We used transcriptome data to identify genes involved in nitrate transport, ammonium transport, amino acid transport, nitrate reduction, ammonium metabolism, and nitrogen regulation. We then analyzed how the expression levels of these genes are related to nitrogen content in roots, stems, and leaves (Figure 4D). In roots, lower total nitrogen (TN) and nitrate nitrogen (NN) levels, and higher ammonium nitrogen (AN) levels, are linked to specific nitrate transporters (NRT1.1, NRT1.2, NRT1.6, and NRT2.7), ammonium transporters (AMT1.1, AMT3.1), amino acid transporters (AAP3, AAP6, and LHT1), glutamate synthase (GS‐N1), glutamine synthetase (GOGAT1, Fe‐GOGAT2), and nitrogen regulation genes (NAC48). In stems, nitrogen regulation involves nitrate and ammonium transporters and amino acid transporters. TN and NN levels are negatively correlated with NRT1.2, NRT1.5, NRT2.6, NRT3.1, NRT3.2, AMT3.1, AAP6, and LHT1, while these genes are positively correlated with AN. In leaves, reductions in NN or increases in AN are associated with NRT1.1, NRT1.6, NRT1.8, NRT2.5, NRT3.2, AMT1.1, AMT1.2, AMT3.1, GS‐N1, Fe‐GOGAT, NLP7, NLP14, NLP41, and NLP48. Fumigation‐induced changes in gene expression and nitrogen distribution are most significant in roots, where numerous nitrogen transporter and metabolism genes are upregulated, leading to notable changes in nitrogen levels. Leaves also exhibit substantial gene expression changes related to nitrogen metabolism, while stems show minimal alterations. This suggests that roots play a critical role in nitrogen regulation, followed by leaves, making them essential targets for improving nitrogen utilization in crops.

### Key factors of nitrogen distribution following DZ fumigation

**FIGURE 5:**
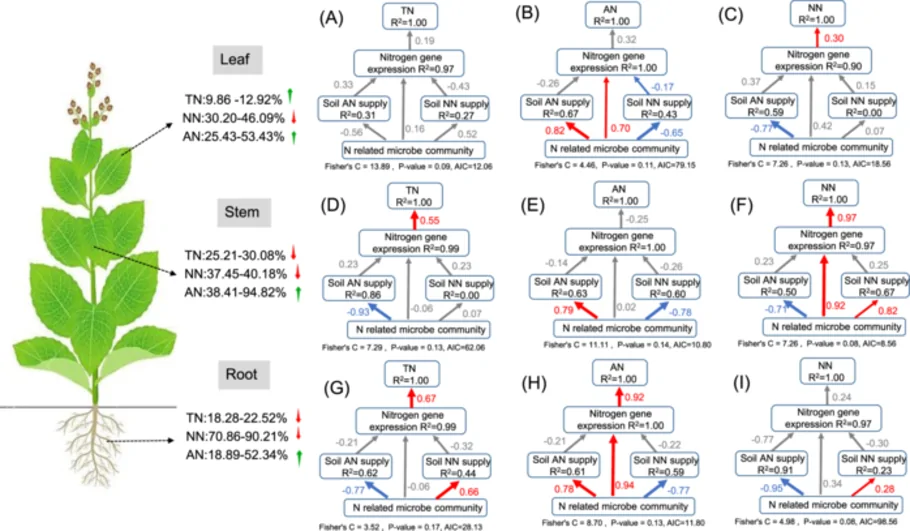
Path diagrams illustrating the relationships between soil microbial communities and nitrogen distribution across different plant compartments. Panels (A)–(C) represent total nitrogen (TN), ammonium nitrogen (AN), and nitrate nitrogen (NN) in leaves; panels (D)–(F) show TN, AN, and NN in stems; and panels (G)–(I) depict TN, AN, and NN in roots. Blue arrows indicate negative relationships, while red arrows indicate positive relationships. Standardized path coefficients are displayed next to the arrows, with arrow size proportional to the strength of the coefficients. Blue or red coefficients denote significant relationships (*p* < 0.05), while gray arrows and coefficients indicate non‐significant relationships (*p* > 0.05). The conditional *R*² values indicate the proportion of variance explained by each variable. The percentage changes in TN, AN, and NN in roots, stems, and leaves under the fumigation treatment compared to the control group are indicated, with increases and decreases represented by upward and downward arrows, respectively.

Fumigation significantly impacted nitrogen‐cycling microorganisms and functional genes, leading to the formation of the “N‐related microbe community” and “Nitrogen gene expression” variables for SEM analysis (Table S2). The SEM results indicated that the nitrogen‐related microbial community exerts a crucial regulatory effect on soil ammonium and nitrate nitrogen levels, with this influence being particularly significant in the roots. Specifically, the community enhances ammonium nitrogen accumulation while reducing nitrate nitrogen levels, likely through the modulation of nitrification and denitrification processes (Figure 5G−I). Conversely, the community also had a negative impact on soil ammonium nitrogen and a positive influence on soil nitrate nitrogen, altering total nitrogen and nitrate nitrogen levels in roots, stems, and leaves, with a less pronounced effect on leaves. Additionally, nitrogen gene expression was directly regulated by the N‐related microbe community rather than by the levels of ammonium and nitrate nitrogen in the soil (Figure 5B,F,H).

The overall effect analysis revealed that the N‐related microbe community had a negative regulatory effect on total nitrogen levels across compartments but a positive effect on nitrate nitrogen, particularly in stems compared to roots and leaves (Figure S4). Moreover, the community positively influenced ammonium nitrogen levels in roots and leaves, with a stronger effect in roots while exerting a minor negative impact on stems. These results showed that DZ fumigation significantly altered the structure of the nitrogen‐cycling microbial community, which in turn affected the availability of ammonium and nitrate nitrogen in the soil. This alteration led to changes in nitrogen transport and metabolism gene expression, ultimately influencing nitrogen distribution in rhizomes and leaves.

## 3 DISCUSSION

### DZ fumigation stimulates soil nitrogen‐cycling microbial community growth

DZ fumigation significantly enhanced the recruitment and colonization of specific microorganisms. Notably, among nitrogen‐cycling microorganisms, denitrifiers such as Sphingomonas and functional genes associated with denitrification, including *nirS, nosZ, nirK, nar*, and *norB*, exhibited a marked increase following fumigation (Table S3). This was accompanied by a substantial rise in denitrification intensity (Figure 3F), indicating that the fumigation significantly stimulated the denitrification process in the soil. Other fumigants, such as chloropicrin, 1,3‐dichloropropene, and dimethyl disulfide, also stimulate denitrifying microorganisms to varying degrees [8, 30]. In an earlier study, we found that DZ fumigation temporarily reduced the abundance of denitrification functional genes (*napA, narG, nirK, nirS, cnorB, qnorB*, and *nosZ*), especially in acidic red soil, when no crops were planted [15]. In the current study, during the later stages of fumigation, the abundance of these genes in soils with planted crops was significantly higher than in control soils (Figure 3G), suggesting that the presence of crops promoted microbial succession after fumigation. For example, after chloropicrin fumigation, soil bacteria in tomato fields recovered faster compared to non‐planted fields [31]. This indicates that fumigation‐induced microbial community changes can evolve to support crop growth.

Three key mechanisms likely contribute to the stimulation of denitrifying microorganisms by DZ fumigation: (1) Increased availability of organic matter: The death of fumigation−sensitive microorganisms releases a large amount of soluble protein, promoting rapid microbial growth. Post−fumigation, soluble amino acids in the soil increased by 1.99–2.32 times [15, 32]. Additionally, the fumigant itself serves as a carbon source. For example, the degradation half‐life of methyl isothiocyanate ranges from several hours to several days, with biodegradation accounting for 80%−90% of total degradation [33]. (2) Reduced competitive pressure: Fumigation kills or inhibits many pathogenic and competitive microorganisms [34], reducing pathogens through antibiotic production or other inhibitory substances, which in turn benefits denitrifiers. Additionally, DZ fumigation reduces the abundance of nitrifiers, such as ammonia‐oxidizing bacteria (AOB) and ammonia‐oxidizing archaea (AOA), which convert ammonia (NH₃) or ammonium (NH₄⁺) to nitrite (NO₂⁻), where higher nitrite concentrations inhibit denitrifier activity [35]. (3) Enhanced resilience of the microbial community: After fumigation, the microbial community begins to recover, with adaptable microorganisms, such as denitrifiers, rapidly reoccupying ecological niches. Studies show that denitrifiers exhibit stronger resistance and resilience to fumigation‐induced changes compared to nitrifiers [36, 37].

### Mechanism of nitrogen distribution driven by soil microorganisms

Fumigation significantly disrupts soil microbial communities, altering ecological functions by suppressing ammonia‐oxidizers and promoting denitrifiers. This shift causes an accumulation of ammonium nitrogen and a reduction in nitrate nitrogen, disrupting the balance of soil nitrogen cycling (Figure 3E,F). Fumigation‐induced disruption of soil nitrogen balance significantly altered nitrogen uptake and distribution in plant tissues, including roots, stems, and leaves (Figure 5). This reallocation of nitrogen was closely associated with the differential expression of key nitrogen metabolism and transporter genes [38]. The expression of multiple genes linked to nitrogen transport and assimilation was notably elevated. These include genes associated with nitrogen uptake, transport, and metabolism, which collectively indicate an enhancement of nitrogen utilization within the crop system (Figure 4D). This shift reflects significant changes in nitrogen‐related processes at the genetic level. These molecular changes likely underlie the observed shifts in nitrogen distribution, highlighting the complex regulatory mechanisms governing plant nitrogen homeostasis in response to soil fumigation [39].

**FIGURE 6:**
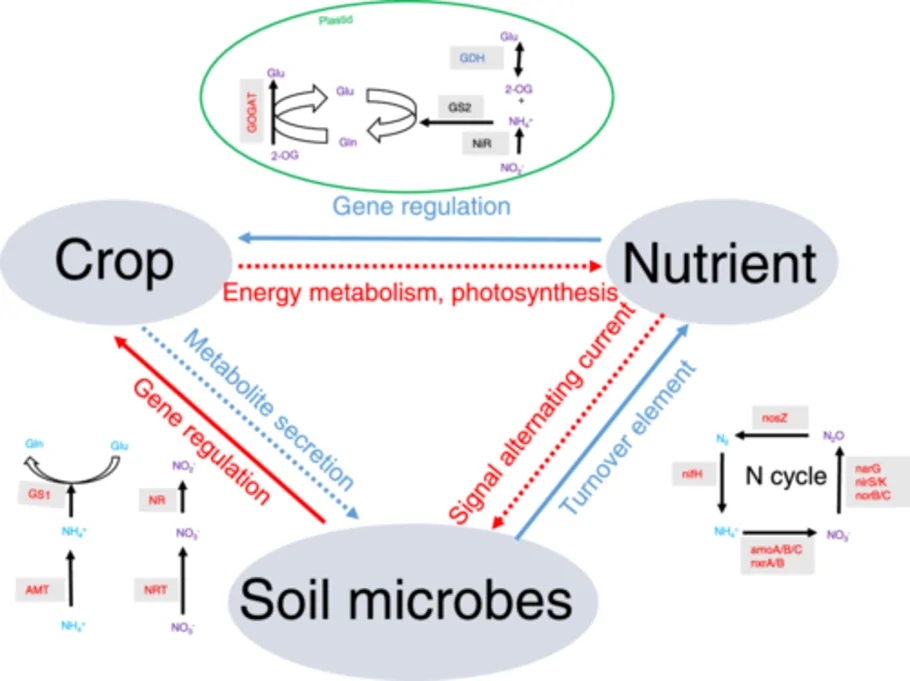
Conceptual model of crop‐microbial‐nutrient interactions. Fumigation alters microbial community structure, which impacts soil nutrient turnover and changes nutrient types and levels. These alterations influence crop gene expression and nutrient uptake. In response, crops regulate nutrient distribution through photosynthesis, which indirectly affects microbial activity. Additionally, crops can directly modify microbial communities through root exudates, further influencing soil nutrient dynamics.

The redistribution of nitrogen in above‐ground plant tissues following fumigation is largely attributable to significant shifts in the expression of nitrogen metabolism genes across roots, stems, and leaves. Notably, nitrate nitrogen, which crops readily absorb, also serves as a signaling molecule that stimulates the expression of nitrogen transporter genes in roots, thereby regulating the uptake and translocation of nitrogen [40, 41]. Our structural equation model indicated that, although direct regulation of gene expression by soil nitrogen was not observed, changes in the soil microbial community exerted a significant influence on the expression of nitrogen metabolism genes in above‐ground tissues (Figure 5). This highlights the pivotal role of soil microorganisms in mediating nutrient uptake and transport in crops. Microbial communities are essential to soil nutrient cycling, and alterations in soil nutrient content and form can directly impact crop gene expression, subsequently affecting nutrient uptake and metabolism (Figure 6). Additionally, crops can modulate microbial signaling through photosynthetic resource allocation and directly influence microbial community composition via root exudates [23].

Fumigation modifies crop‐microbe interactions through several mechanisms [42, 43]: (1) It enhances the decomposition of organic matter and the mineralization processes mediated by soil microorganisms, thereby increasing the availability of key nutrients, such as soluble amino acids, phosphorus, and nitrogen, for plant uptake. (2) Fumigation improves soil structure, thereby optimizing the root environment and enhancing the efficiency of water and nutrient absorption, which is critical for the translocation of nutrients to above‐ground tissues, as root health directly influences shoot growth. (3) fumigation‐induced changes in microbial community structure may stimulate the production of crop hormones, including auxins, cytokinins, and gibberellins, which regulate root development, shoot growth, and nutrient transport. These mechanisms collectively enable crops to more efficiently utilize soil resources, optimize nutrient distribution within above‐ground tissues, and enhance overall plant growth and yield. However, further research is necessary to fully understand the complex interactions among soil microbes, plant endophytes, and nutrient metabolism, with isotope labeling being a particularly valuable tool for such studies.

## 4 CONCLUSION

Dozamet soil fumigation led to an increase in ammonium nitrogen and a decrease in nitrate nitrogen across crop roots, stems, and leaves, significantly enhancing nitrogen metabolism. Fumigation‐induced alterations in microbial communities, specifically the reduction in ammonia‐oxidizers and increase in denitrifiers, drive these changes. These shifts disrupt nitrogen turnover, increasing ammonium and decreasing nitrate levels, which in turn imbalances nitrogen supply to the rhizosphere and hampers the crop's inorganic nitrogen uptake Additionally, changes in microbial communities impact crop nitrogen metabolism and the expression of transporter genes, thereby facilitating more effective nitrogen transport and utilization from the soil to the above‐ground crop parts. Our study elucidates the linkage between soil nitrogen dynamics and crop nitrogen metabolism, offering new perspectives for optimizing crop nutrient management.

## 5 METHODS

### Soil sample preparation and fumigation

Soil samples were collected from Fangshan, Beijing (39.7077° N, 115.9605° E), representing alkaline fluvo‐aquic soil, and from Wenshan, Yunnan (23.3681° N, 104.2493° E), representing acidic red soil. The physicochemical properties of these soils are detailed in Table S1. The collected soil was sieved through a 2 mm mesh and adjusted to an absolute water content of 13%. Before the experiment, the soil was supplemented with sodium nitrate, monopotassium phosphate, and potassium chloride to achieve final concentrations of 200 mg kg^−1^ N, 150 mg kg^−1^ P, and 150 mg kg^−1^ K. The soil was pre‐incubated in the dark at 25°C for 1 week.

For fumigation, dazomet (98% granules, Nantong Shizhuang Chemical Co., Ltd., Jiangsu) was mixed with the soil at a rate of 150 mg/kg. The soil was then fumigated in a desiccator sealed with petroleum jelly to ensure airtightness and incubated at 28°C in the dark for 7 days. A control group without dazomet was also set up. After fumigation, the remaining fumigant was vented in a fume hood. Soil samples were collected and stored at 4°C and −80°C (designated as Day 0 post‐fumigation) for the determination of soil chemical parameters and microbial indicators.

### Tobacco transplantation and sample collection

*Nicotiana benthamiana* seeds (provided by the Tobacco Research Institute of the Chinese Academy of Agricultural Sciences) were sown in plastic culture boxes. When the seedlings reached the three‐leaf stage, uniformly growing seedlings were selected. Their roots were washed with sterile water before transplanting into fumigated and non‐fumigated soils. Tobacco was transplanted into individual pots, each containing 1 kg of soil. The pots were placed in a greenhouse under controlled conditions: 28°C temperature, 60% relative humidity, and a 12‐h light/12‐h dark photoperiod Sampling was conducted on Days 30 and 50 post‐fumigation. For tobacco sample collection, the entire crop was uprooted, and roots, stems, and leaves were washed with distilled water and dried. The roots, stems, and leaves were separated; some samples were dried at 55°C to constant weight for the determination of dry mass and total nitrogen. Other samples were placed in pre‐chilled centrifuge tubes, flash‐frozen in liquid nitrogen for 5 min, and stored at −80°C for the determination of inorganic nitrogen concentrations, nitrogen metabolism enzyme activities, and transcriptome analysis. For rhizosphere soil sampling, loosely adhered soil was shaken off, and tightly adhered soil was brushed off and collected; some samples were used for soil nitrogen transformation assays, and others for metagenomic sequencing.

### Soil nitrogen‐related parameters determination

Soil physicochemical properties were determined following standard methods [44]. Available phosphorus was extracted with NaHCO_3_ and analyzed using a flow analyzer; available potassium was extracted with CH_3_COONH_4_ and analyzed using a flame photometer; pH was determined using a 2.5:1 water−soil ratio with a potentiometer; electrical conductivity was measured using a 2.5:1 water−soil ratio; organic matter was determined using the potassium dichromate titration method. Nitrate nitrogen and ammonium nitrogen were extracted with KCl and measured using a flow analyzer.

The potential of denitrification was measured by incubating 10 g of fresh soil in 20 mL headspace vials filled with deionized water, sealed, and incubated in the dark at 28°C. Nitrate nitrogen concentrations were determined at 0 and 24 h using the KCl extraction method, and denitrification intensity was calculated as nitrate nitrogen consumption per unit time using the following Equation (1):

(1) $$V_{d}=\frac{C_{0}\left({{\text{NO}}_{3}}^{-}-N\right)-C_{t}\left({{\text{NO}}_{3}}^{-}-N\right)}{24\;h}(\mathrm{mg\cdot}{\text{kg}}^{-1}\cdot h^{-1}).$$

The potential of nitrification was measured by incubating 10 g of fresh soil in 20 mL headspace vials sealed with sterile cotton, incubated in the dark at 28°C, and ammonium nitrogen concentrations were determined at 0 and 24 h using the KCl extraction method. Nitrification intensity was calculated as ammonium nitrogen consumption per unit time using the following Equation (2):

(2) $$V_{n}=\frac{C_{0}\left({{\text{NH}}_{4}}^{+}-N\right)-C_{t}({{\text{NH}}_{4}}^{+}-N)}{24\;h}(\mathrm{mg\cdot}{\text{kg}}^{-1}\cdot h^{-1}).$$

The supply rates of ammonium and nitrate nitrogen in the rhizosphere were calculated using the crop rhizosphere available nitrogen supply models (3) and (4).

(3) $$V_{{{\text{NH}}_{4}}^{+}}=\frac{F_{m{{\text{NH}}_{4}}^{+}}\times C_{r{{\text{NH}}_{4}}^{+}}\times r}{K_{m{{\text{NH}}_{4}}^{+}}+C_{r{{\text{NH}}_{4}}^{+}}}-V_{n},$$

(4) $$V_{{{\text{NO}}_{3}}^{-}}=\frac{F_{m{{\text{NO}}_{3}}^{-}}\times C_{r{{\text{NO}}_{3}}^{-}}\times r}{K_{m{{\text{NO}}_{3}}^{-}}+C_{r{{\text{NO}}_{3}}^{-}}}+V_{n}-V_{d}.$$

$V_{{{\text{NH}}_{4}}^{+}}$ and $V_{{{\text{NO}}_{3}}^{-}}$ represent the supply rate of ammonium nitrogen and nitrate nitrogen in the rhizosphere (mg·kg^−1^·h^−1^), respectively. *C*_r_ denotes the concentration of ammonium nitrogen and nitrate nitrogen in the rhizosphere soil (mg·kg^−1^·h^−1^). $V_{n}$ and $V_{d}$ refer to the nitrification and denitrification rates, respectively (mg·kg^−1^·h^−1^). The parameter r is set at 0.1 mm, representing the root radius for nitrogen absorption. *F*_m_ and *K*_m_ indicate the maximum inflow rates of ammonium nitrogen and nitrate nitrogen into roots, and the conduction consumption constants of ammonium nitrogen and nitrate nitrogen in soil, respectively [45].

### Plant nitrogen and enzyme activity determination

Inorganic nitrogen content in roots, stems, and leaves was determined by grinding the samples in liquid nitrogen, extracting with KCl, and measuring using a flow analyzer. Total nitrogen was measured by drying and pulverizing the samples and analyzing 50 mg of the sample using an elemental analyzer in CN mode.

Protein content in roots, stems, and leaves was extracted, and nitrate reductase (NR), nitrite reductase (NiR), glutamine synthetase (GS), and glutamate synthase (GOGAT) activities were measured using a BCA protein assay kit (Thermo Scientific) and reading absorbance at 450 nm.

### Transcriptome sequencing and analysis

Total RNA from roots, stems, and leaves was extracted using the RNAprep Pure Plant Kit and checked for quality and purity using agarose gel electrophoresis and Nanodrop 2000. High‐quality RNA was used to synthesize cDNA libraries, which were constructed through end repair, A‐tailing, adapter ligation, and PCR amplification. Sequencing was performed on an Illumina NovaSeq. 6000 platform (Shanghai Majorbio Bio‐Pharm Technology Co., Ltd.). Raw data were filtered using fastp (v0.20.0) to remove reads with adapters, reads containing more than 10% unknown bases (N), and reads with low quality (*Q*‐value ≤ 20) to ensure the accuracy of subsequent analyses. Clean reads were then quality‐checked using FastQC (v0.11.9). Contig assembly was performed using Trinity (v2.11.0) to obtain unigenes, applying default parameters with a minimum contig length of 200 bp. Gene annotations were carried out by aligning the unigenes with protein databases, including NR, Swiss‐Prot, and KEGG, using BLASTX with an e‐value cutoff of 1e^−5^. RSEM (v1.3.3) was employed to calculate gene expression levels, and DESeq. 2 (v1.28.1) was used to identify differentially expressed genes with a threshold of |log_2_ fold change| ≥ 1 and adjusted *p*‐value < 0.05. These analyses were conducted to explore transcriptomic characteristics and functional responses under different treatments.

### Metagenomic sequencing and analysis

Total soil DNA was extracted using the E.Z.N.A.® Soil DNA Kit and checked for quality using agarose gel electrophoresis and a spectrophotometer (Nanodrop 2000). Quality‐controlled DNA was fragmented, end‐repaired, adapter‐ligated, and PCR‐amplified to construct sequencing libraries, which were sequenced on an Illumina NovaSeq platform (Shanghai Majorbio Bio‐Pharm Technology Co., Ltd.). Raw data were quality‐checked using fastp (v0.20.0) to obtain the clean data, which were then assembled using MEGAHIT (v1.1.2) with default parameters for metagenome assembly. Gene prediction was performed using MetaGeneMark (v3.38), and a nonredundant gene set was constructed by clustering genes at 95% identity using CD‐HIT (v4.6). For functional and taxonomic annotation, the amino acid sequences of the nonredundant gene set were compared against protein databases, including NR, eggNOG, KEGG, and CAZy, using DIAMOND (v0.9.24) with an e‐value cutoff of 1e^−5^. The species‐level annotation was based on the alignment results with the NR database, and functional annotations were derived from the KEGG, eggNOG, and CAZy databases. The abundance of functional genes was calculated and analyzed to understand their roles in nutrient cycling and soil microbial community dynamics under different treatments.

### Statistical analysis

Tukey's ANOVA was used to analyze the significance of change of total nitrogen, ammonium nitrogen, and nitrate nitrogen in different parts of root, stem, and leaf after fumigation. The correlation between these nitrogen forms and spatial location was calculated using Spearman's correlation. Heatmap of correlation between nitrogen and enzyme activity conducted by Hmisc package in R (4.4.1). GO enrichment analysis was performed using Goatools, and significant GO functions were identified with the Fisher test. Significant differences in abundance between the fumigation treatment and control groups were identified using Linear discriminant analysis Effect Size differential discriminant analysis, based on the non‐parametric factorial Kruskal−Wallis sum‐rank test. Linear discriminant analysis (LDA) was then applied to estimate the influence of each species' abundance on the differential effect, with the LDA threshold set at greater than 3.5. Structural equation models (SEM) were used to compare the direct and indirect effects of the soil microbial community on nitrogen distribution in different compartments using the piecewiseSEM package in R (4.4.1).

## AUTHOR CONTRIBUTIONS

**Wensheng Fang**: Conceptualization; supervision; investigation; methodology. **Wenfeng Tian**: Data curation; writing—original draft; writing—review and editing. **Dongdong Yan**: Investigation. **Yuan Li**: Formal analysis. **Aocheng Cao**: Writing—review and editing. **Qiuxia Wang**: Funding acquisition; writing—review and editing.

## CONFLICT OF INTEREST STATEMENT

The authors declare no conflicts of interest.

## ETHICS STATEMENT

No animals or humans were involved in this study.

## Supporting information


**Figure S1:** Microbial community diversity and functional diversity.
**Figure S2:** Characteristics of soil nitrogen transformation and microbial response on the 0th day.
**Figure S3:** GO enrichment analysis.
**Figure S4:** Standardized total effects (direct plus indirect effects) calculated by the SEM.

**Table S1:** Physical and chemical properties of the tested soils.
**Table S2:** Construction of component variables for SEM.
**Table S3:** N related gene abundance and the statistical parameter.

## Data Availability

The data that support the findings of this study are available from the corresponding author upon reasonable request. The raw data of transcriptome (CRA021448, https://bigd.big.ac.cn/gsa/browse/CRA021448) and metagenome (CRA021339, https://bigd.big.ac.cn/gsa/browse/CRA021339) in this study have been deposited in the Genome Sequence Archive in National Genomics Data Center, China National Center for Bioinformation, Beijing Institute of Genomics, Chinese Academy of Sciences. Supplementary materials (figures, tables, graphical abstract, slides, videos, Chinese translated version, and update materials) may be found in the online DOI or iMeta Science http://www.imeta.science/imetaomics/.
