# Supplementary material for: Linkages between soil nutrient turnover and above‐ground crop nutrient metabolism: The role of soil microbes
Source: IMetaOmics. 2025 Feb 3;2(1):e55. doi: 10.1002/imo2.55 (PMC12806472; doi:10.1002/imo2.55)
Supplement: Supplementary file 1 — Figure S1: Microbial community diversity and functional diversity. Figure S2: Characteristics of soil nitrogen transformation and microbial response on the 0th day. Figure S3: GO enrichment analysis. Figure S4: Standardized total effects (direct plus indirect effects) calculated by the SEM. [file IMO2-2-e55-s001.docx]

**Supporting information to：**

**Linkages between soil nutrient turnover and above-ground crop nutrient metabolism: the role of soil microbes**

**Running title: microbial role in soil nutrient turnover and crop nutrition**

Wensheng Fang^1^, Wenfeng Tian^1,2^, Dongdong Yan^1^, Yuan Li^1^, Aocheng Cao^1^, Qiuxia Wang^1*^

^1^State Key Laboratory for Biology of Plant Diseases and Insect Pests, Institute of Plant Protection, Chinese Academy of Agricultural Sciences, Beijing 100193, China

^2^Horticulture College, Hunan Agricultural University, Changsha 41028, China

Correspondence: [wqxcasy@163.com](mailto:wqxcasy@163.com) (Qiuxia Wang)

**Figure S1** Microbial community diversity and functional diversity. (A) Shannon diversity index for taxonomic classification at the bacterial genus level. (B) Functional Shannon diversity index based on KEGG KO level. (C) Principal Component Analysis (PCA) of bacterial taxonomy at the species level, and (D) PCA based on functional KEGG KO level.

**Figure S2** Characteristics of soil nitrogen transformation and microbial response on the 0th day. (A) Ammonium nitrogen (AN), (B) Nitrate nitrogen (NN). (C) Overview of functional gene changes related to the nitrogen cycle pathway. Colored lines represent different pathways, with genes responsible for each pathway marked along the lines. Gene names are labeled red for significant up-regulation compared to the control, blue for significant down-regulation, and black for no significant change. Asterisks indicate the significance level. (D) LEfSe multi-level species hierarchy diagram. Different colored nodes indicate microbial groups significantly enriched in the respective treatment group, contributing to group differences. Light yellow nodes represent microbial groups with no significant difference or effect between groups. (E) LDA discriminant histogram. Higher LDA scores indicate a greater influence of species abundance on the differential effect. Identified marked microbial abundance changes in the 0th day (F) and the 50th day (G).

**Figure S3** GO enrichment analysis. (A) Root, (B) Stem, (C) Leaf. The vertical axis represents the GO terms, and the horizontal axis represents the Rich factor, which is the ratio of the number of genes enriched in each GO term to the number of annotated genes in the background. A larger Rich factor indicates a higher degree of enrichment. The size of the dots represents the number of genes in each GO term, and the color of the dots corresponds to different Padjust ranges.

 **Figure S4** Standardized total effects (direct plus indirect effects) calculated by the SEM.
